# The Problem with Academic Medicine: Engineering Our Way into and out of the Mess

**DOI:** 10.1371/journal.pmed.0020111

**Published:** 2005-04-26

**Authors:** Jonathan Rees

## Abstract

Medical schools have come to resemble schools of molecular biology, while medical research has become focused on molecules rather than patients

*“My advice is to go for the messes—that's where the action is.”—Steven Weinberg [1]*

Academic medicine has tried to avoid the mess that has characterised much of clinical practice over the last quarter century. Instead its success has come from using techniques developed in biochemistry and molecular genetics, and applying them to disease. It has been a highly productive approach. For example, identification of the clinical syndrome AIDS, isolation of the causative agent, and development of diagnostic testing and effective therapy all happened in the space of a few years. Alongside such successes has come a redefinition of academic medicine. Medical research has become biomedical research; discovery flows from bench to bedside and if it stutters, an injection of funds for more effective translation is required [2]. A plethora of organisms are sequenced, and all the genes in the mouse are to be “knocked out” [3]—all to serve the goal of improving human health, so we are told.

Even the idea of clinical disciplines appears slightly passé, their traditional names a throwback to a less scientific age—dermatology is now dermatological science, and neurology and psychiatry are now clinical neuroscience [4]. And if you are tempted at the end of the clinic—before you return to the sanctuary of the lab—to wonder about the mess of clinical medicine, to ask how it all fits together, and to question the real outcomes of what you do, you run the risk of being put down. The put down goes like this: we do science; we do not question whether “Coke is better than Pepsi or approach A is better than approach B” [5]; we leave such choices to the consumer and the HMOs to decide upon. Back to the mice, and future tenure.

## Clinical Medicine: The Missing Subject

A striking aspect of the modern medical research university is the relative absence of clinical medicine from its portfolio of activities. Just look at what our successors are being encouraged to study. Most students will spend three or more years studying subjects such as cell biology, genetics, stem cell biology, or biochemistry, with a small remnant learning some clinical epidemiology and trials methodology. Now take yourself back to the clinic, spend a week seeing patients, and ask what academic establishment is needed to improve health care? Well, of course, some cell biology and the like are needed, but I suggest such disciplines should account for only 20% of our effort.[Fig pmed-0020111-g001]

**Figure pmed-0020111-g001:**
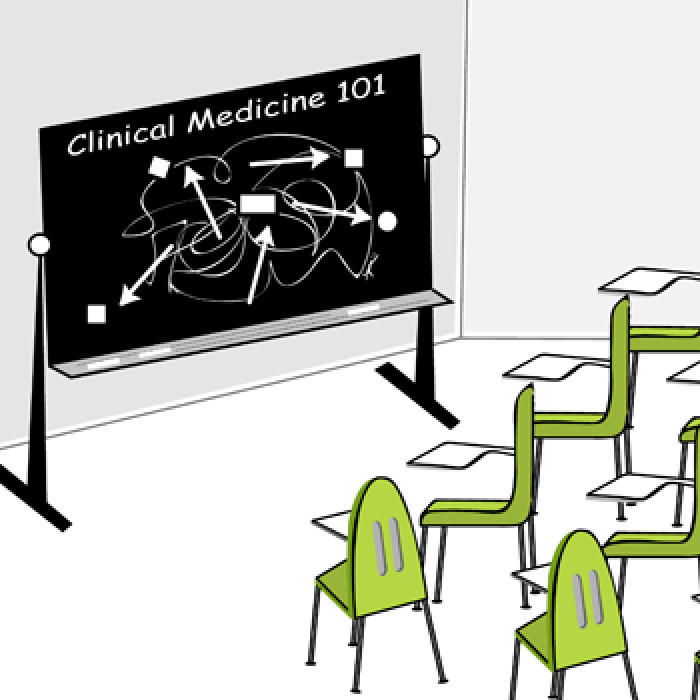
A striking aspect of the modern medical research university is the relative absence of clinical medicine from its portfolio of activities (Illustration: Sapna Khandwala, Public Library of Science)

The rest of our effort should be directed towards operations research; the basis of expertise and decision-making processes; informatics, from the mundane issue of paper versus digital clinical records to the representation of clinical information in ways that are helpful for both doctor and patient; health-care policy, determining why effective therapies are so often not available, and why some diseases are researched more than others; and psychology, determining why patients' worldviews are so often different from ours and whether cognitive limitations (by either health professionals or patients) can account for some of these differences. Underpinning all of this should be the basic medical sciences: economics, statistics, and physiology.

## Academic Kudos Versus Improving Health

So why are our institutions not fit for the purpose of improving patients' health? Herbert Simon, the polymath and Nobel laureate in economics, observed many years ago that medical schools resembled schools of molecular biology rather than of medicine [6]. He drew parallels with what had happened to business schools. The art and science of design, be it of companies or health care, or even the type of design that we call engineering, lost out to the kudos of pure science. Producing an economics paper densely laden with mathematical symbols, with its patently mistaken assumptions about rational man, was a more secure way to gain tenure than studying the mess of how real people make decisions.

The same problem also applies to medicine. Flushed with success from the golden age of medical discovery, a marriage was brokered between a simple and mistaken model of medical discovery in which there was a unidirectional flow of information from bench to clinic, and the safety and control afforded by the technical facility of modern biology. Once in place, the forces needed to sustain this ill-judged union were not difficult to recognise.

Most medical researchers rarely, if ever, see patients. Most who argue for the necessity of the pyramid of discovery, with biochemistry and genetics at the base and clinical research at the apex, work at the base themselves [2]. However, many of the major discoveries that have had a direct impact on clinical practice arose from clinical disciplines rather than from generic biomedical approaches—consider hip replacements, cataract surgery, the importance of Helicobacter pylori, phototherapy, in vitro fertilisation, and minimally invasive surgery [4]. In the biomedical model, these successes are brushed aside as being of historical interest only. From finding genes to gene therapy and stem cell therapy, both the public and research community itself are fed a biased view of medical advances. Cancer cured (in mice) again.

## Medicine: A Science of the Artificial

A number of arguments suggest that the conventional wisdom that has underpinned the institutions of academic medicine is breaking down. First, the pace of major clinical advance, despite increases in funding, is slowing [2]. Second, there is increased external scrutiny (from many sources) of medicine, not least because of the large increases in the cost of health care. Third, and a useful indicator that something is amiss, a career in academic medicine is increasingly unattractive—the new generation of students clearly see the academic towers as ill suited to solving the problems that they experience in the clinic.

## The Two Epistemologies That Underpin Clinical Advance

There have been two intellectual movements in the last half century influencing medical practice. The first—the enormous evolution of technological facility in biochemistry and genetics—needs little comment as its influence is all around. The second, still inchoate, is the attempt to elucidate the laws that govern clinical practice. It is to the great credit of the clinical epidemiology movement that it focussed attention once again on how medicine was practiced. No longer was medicine just the application of theories developed in the laboratory, but there was the glimmering of an intellectually coherent discipline centred on diagnosis and intervention. When seen alongside the now classical work of Wennberg, showing the remarkable variation in the delivery of health care across populations [7], medicine could be seen in a new light. No longer was medicine a mere application of “basic” science, of lessons from physiology, but, instead, medicine was an artefact of engineering—health and medical care was something we build, something we design. Just as informatics and computing are not solely about building faster chips, but about how information is ordered and represented, and how machines and humans interact; the same is true for medicine. To use Herbert Simon's phrase, medicine is a “science of the artificial”, *artificial* as in artificial intelligence: an artefact or something created for a purpose [6].

## The Codification of Medicine: Bringing Order to the Mess

The principal intellectual question of the next quarter century for academic medicine is to what extent medicine is capable of codification. How and to what degree can we codify—produce rules—of how decisions in medicine should be made. The march of industrial progress for the last 200 years has been the replacement of implicit knowledge and experience by formal teaching, rule-based behaviour, specialisation, and the division of labour. How will the mess that is clinical practice be ordered by this new epistemology?

How do the results of clinical trials inform the therapy of an individual patient? Despite the views of many, how to interpret numerical data as evidence to guide action in a particular case has troubled the greatest mathematicians for centuries [8]. How do we assimilate information of different kinds? What is the relative proportion of local factors versus summary measures in the treatment of a patient? The answers are not clear. Trials are expensive, infrequent, and remote from much of clinical care. Often the debate seems to resemble some medieval battle, with artillery and army being towed into place to siege a recalcitrant city—think, for example, of the intense debate surrounding the value of mammography [9]. By contrast, clinical practice seems to resemble more guerrilla warfare, where the lie of the local terrain trumps any fickle general predictions. The set of statistical techniques developed for use in agriculture by the great R. A. Fisher and others hardly seems appropriate for the questions we wish to address [10]. Populist ideas about evidence, and the weight of evidence—the pseudoscience that adorns so many guidelines—is almost too embarrassing to mention.

I mentioned the issue of cost with good reason. We have gotten used to pretending that cost is not an issue when new treatments are being researched. Medicine is as much engineering as science, and what needs to be rewarded in medical research is the ability to innovate within a financial envelope. Being able to invent solutions at an affordable price is a design constraint: one that needs to be seen as a challenge. The solution “at any price” is lazy: cheap PCs are what made the World Wide Web so revolutionary.

## Conclusion: Coke Versus Pepsi Revisited

Codification reminds us that medicine makes heavy demands on its practitioners, demands that in all too many cases can be usefully mitigated with appropriate information systems. Here the question is how we can design systems that record clinical information in a way that facilitates direct clinical care, and feeds back and informs clinical discovery. The danger is to believe that such an advance is not an intellectual problem, but just a mere choice of which brand of software to use for clinical records. There is no greater example of academic medicine's hubris than imagining that designing medical practice is like choosing between “Coke or Pepsi” [5]. It is like saying Tim Berners Lee's invention of the World Wide Web was on a par with inventing another typeface.
